# A seed resource for screening functionally redundant genes and isolation of new mutants impaired in CO_2_ and ABA responses

**DOI:** 10.1093/jxb/ery363

**Published:** 2018-10-20

**Authors:** Felix Hauser, Paulo H O Ceciliato, Yi-Chen Lin, DanDan Guo, J D Gregerson, Nazia Abbasi, Diana Youhanna, Jiyoung Park, Guillaume Dubeaux, Eilon Shani, Nusra Poomchongkho, Julian I Schroeder

**Affiliations:** 1 Division of Biological Sciences, Cell and Developmental Biology Section, University of California San Diego, La Jolla, CA, USA; 2 College of Pharmacy, Second Military Medical University, Shanghai, China; 3 School of Plant Sciences and Food Security, Tel Aviv University, Ramat Aviv, Israel

**Keywords:** Arabidopsis, artificial microRNA, forward genetic screen

## Abstract

The identification of homologous genes with functional overlap in forward genetic screens is severely limited. Here, we report the generation of over 14000 artificial microRNA (amiRNA)-expressing plants that enable screens of the functionally redundant gene space in Arabidopsis. A protocol was developed for isolating robust and reproducible amiRNA mutants. Examples of validation approaches and essential controls are presented for two new amiRNA mutants that exhibit genetically redundant phenotypes and circumvent double mutant lethality. In a forward genetic screen for abscisic acid (ABA)-mediated inhibition of seed germination, amiRNAs that target combinations of known redundant ABA receptor and *SnRK2* kinase genes were rapidly isolated, providing a strong proof of principle for this approach. A new ABA-insensitive amiRNA line that targets three *avirulence-induced gene 2*(*-like*) genes was isolated . A thermal imaging screen for plants with impaired stomatal opening in response to low CO_2_ exposure led to the isolation of a new amiRNA targeting two essential proteasomal subunits, *PAB1* and *PAB2*. The seed library of 11000 T2 amiRNA lines (with 3000 lines in progress) generated here provides a new platform for forward genetic screens and has been made available to the Arabidopsis Biological Resource Center (ABRC). Optimized procedures for amiRNA screening and controls are described.

## Teaser

Over 14000 amiRNA-expressing Arabidopsis plants were generated, to be made publicly available, enabling screening of redundant genes. Known and new genes were identified.

## Introduction

The presence of large gene families in plants, including Arabidopsis (Arabidopsis Genome Initiative, 2000), leads to functional genetic redundancies or partial functional overlap among closely related genes. Functional overlap and partial or complete redundancy between different family members has been proposed to provide a buffer for loss or gain of function mutation events and mechanistic robustness of cellular networks (Wagner, 2005). This is considered to be a main reason for the lack of observable phenotypes in single-gene deletion mutants and increasing severity of phenotypes in higher order mutants of homologous genes (Ma *et al.*, 2009; Park *et al.*, 2009). Identification and characterization of functionally overlapping genes in genetic screens is limited, as is evident from the relatively low number (591 of all Arabidopsis genes) of genes not associated with a single mutant phenotype (Lloyd and Meinke, 2012). Analysis of genome-wide gene family definitions showed that the Arabidopsis genome includes over 22000 genes belonging to gene families (Hauser *et al.*, 2013). Strategies and tools have been developed to enable screens of the functionally redundant gene space. Recently, an artificial microRNA (amiRNA)-based computational design approach was introduced (Hauser *et al.*, 2013). AmiRNAs designed to specifically target diverse combinations of gene family members or combinations of subfamily members enable the screening of partial overlapping homologous gene functions at a genome-wide scale. The presented platform also provides an approach for the capture of homologous gene silencing phenotypes, for which higher order loss of function mutants would lead to lethality, as illustrated by a mutant identified here.

Here, we report the generation of over 11000 T2 amiRNA lines and 3000 additional amiRNA lines by transformation of Arabidopsis Col-0 with a previously published amiRNA library (Hauser *et al.*, 2013) and screening of T2 amiRNA lines for abscisic acid (ABA)-insensitive seed germination phenotypes or plants with low-CO_2_-insensitive high-leaf-temperature phenotypes. Methods are described to identify robust amiRNA mutants and how to avoid pitfalls of this approach. The screen rapidly identified two amiRNAs that target three *PYR*/*RCAR* ABA receptor- (Ma *et al.*, 2009; Park *et al.*, 2009) or six SNF1-related kinase- (*SnRK2*; Mustilli *et al*., 2002; Yoshida *et al.*, 2002; Fujii and Zhu, 2009) encoding genes known to be involved in ABA-mediated control of seed germination. One candidate line that shows an ABA-insensitive seed germination phenotype contains an amiRNA that targets three genes of unknown function, which are annotated as *Avirulence-induced gene 2* (*AIG2A*; AT3G28930), *Avirulence-induced gene 2-like protein A* (*AIG2LA*; AT5G39720), and *Avirulence-induced gene 2-like protein B* (*AIG2LB*; AT5G39730). One amiRNA that causes a low-CO_2_-insensitive high-leaf-temperature phenotype targets two genes encoding proteasomal α2-subunits, annotated as *PAB1* (AT1G16470) and *PAB2* (AT1G79210), for which double mutation causes lethality. New amiRNA lines that target the gene for proteasomal α7-subunit, annotated as *PAG1* (AT2G27027), were constructed resulting in a similar stomatal phenotype. Together these observations indicate a rate-limiting role of the intact proteasome for stomatal opening responses.

## Materials and methods

### Plant material, growth conditions and transformation

Arabidopsis accession Columbia-0 was used as the background for all amiRNA transformations of the library. Surface-sterilized seeds (15 min 70% ethanol, 0.1% sodium dodecyl sulfate; three to four washes with ~100% ethanol; alternatively 10 min 50% bleach, 0.05% Tween-20; four to six washes with water; Lindsey *et al.*, 2017) of Arabidopsis were cold-treated for 2–5 d at 4 °C and germinated on half-strength Murashige and Skoog basal medium supplemented with Gamborg’s vitamins (Sigma-Aldrich (Murashige and Skoog, 1962; Gamborg *et al.*, 1968), 0.8% Phytoagar (Difco, Franklin Lakes, NJ, USA) and pH adjusted (pH 5.8; 2.6 mM MES titrated with potassium hydroxide). After 5–7 d, plants were transferred to plastic pots containing sterilized premixed soil (Sunshine Professional Blend LC1 RS; Sunshine; supplemented with an appropriate amount of insecticide (Marathon, Gnatrol)) and propagated under the following conditions: long day (16 h light/8 h dark); 23–27 °C; 20–70% humidity, 60–100 mmol m^−2^ s^−1^ light.

Plant transformation by floral dip was performed as described elsewhere (Clough and Bent, 1998) with the following modifications. *Agrobacterium tumefaciens* GV3101::pMP90 (Koncz and Schell, 1986) was grown under selection of all markers, i.e. genomic (rifampicin), Ti-plasmid (gentamicin), pSOUP (tetracycline) and T-DNA plasmid (spectinomycin). The infiltration medium for resuspension of the bacteria and floral dip contained 5% sucrose (w/v) and 0.02% (v/v) Silwet L-77 (Clough and Bent, 1998).

Large scale transformation with the amiRNA library pools (Hauser *et al.*, 2013) was performed as described elsewhere (Cutler *et al.*, 2000) with the following modifications. One microgram of DNA of each amiRNA sublibrary (Hauser *et al.*, 2013) was electroporated into a total of 500 µl electrocompetent *A. tumefaciens* cells. The 20 bp and 21 bp amiRNA sublibrary variants for each pool were individually electroporated and combined at this stage. After 2 h at 30 °C in non-selective Luria–Bertani–Miller medium (LB, Teknova), the cells were spread on 20 LB plates (1.5% agar; 150 mm diameter) containing all the appropriate antibiotics (rifampicin, gentamycin, tetracycline, spectinomycin) and grown for 3 d at 30 °C. The bacteria were scraped from the plates, resuspended in 5 ml LB and concentrated by centrifugation for 20 min at 5855 *g*. Plants were transformed by spraying the flowers with this suspension of the bacteria in infiltration medium (adjusted to an optical density at 600 nm of 0.5) twice with 1 week between the treatments. T1 plants were selected on plates supplemented with 75 µM phosphinotricin or directly on soil by spraying diluted herbicide (1000× dilution, Finale^®^; Bayer, Research Triangle Park, NC, USA) four times with 2–7 d between the treatments. Herbicide-resistant plants were transferred to soil and grown to full maturity and T2 seeds collected from individual plants. When appropriate, media for growth of bacteria or plant selection contained the following concentrations of antibiotics (mg ml^−1^): carbenicillin 100, gentamycin 25, kanamycin 30, rifampicin 50, spectinomycin 100, tetracycline 10, and phosphinotricin 15.

### Screen for abscisic acid-insensitive cotyledon emergence phenotype

T2 plants were screened individually for insensitivity of seed germination to ABA in 96-well plates (100 µl 0.1% agarose supplemented with 2 µM (±)-ABA, Sigma-Aldrich). Approximately 10–20 seeds were used from each T2 plant. For the pooled screening, approximately 10–50 seeds of 90 individual T2 plants were mixed, surface sterilized and sprinkled onto agar plates (3 μM (±)-ABA; Sigma-Aldrich). As control for ABA insensitivity, *abi4-1* (ABRC, CS8104) or *abi5-1* (ABRC, CS8105) was used and Col-0 was used as a wild-type control. A putative ABA-insensitive phenotype was scored in a binary manner for similarity to the *abi4-1* or *abi5-1* phenotype and difference from wild-type after 5–8 d using green cotyledons as indicator (Kuhn *et al.*, 2006). For lines that showed a putative ABA insensitivity, the seed germination assay was repeated by propagating individual T2 seedlings to the next generation (T3) and using seeds of the T3 generation for ABA sensitivity assays. This time, seeds were placed on plates with and without ABA (2 µM (±)-ABA; Sigma-Aldrich) and images were taken daily for 7 d and emergence of radicles and cotyledons was counted manually using Fiji (Schindelin *et al.*, 2012). For candidates of the individual screen the T2 seeds were used for the repetition of the germination assay.

For candidates of the pooled screen ABA-insensitive seedlings were transferred to plates containing 75 µM phosphinotricin, and after 7–10 d resistant seedlings were transferred to soil, grown up to full maturity, and the T3 seeds used for the validation of the ABA-insensitive germination phenotype.

### Screen for CO_2_-insensitive leaf temperature phenotype

Seeds of T2 plants were germinated in 96-pot-flats (254 mm×508 mm; East Jordan Plastics, East Jordan, MI, USA) on soil with each pot containing seeds from one plant. After 7 d, seedlings were sprayed with a 1000× dilution of Finale^®^ (Bayer), and 2–3 d later pale seedlings were removed and only one healthy dark green seedling was left per pot. After 19 d under standard growth conditions, the plants were exposed to 150 ppm CO_2_ for 2 h in a Percival growth chamber. A first set of thermal images was taken with a FLIR A320 thermal imaging camera (FLIR, Wilsonville, OR, USA). Subsequently the plants were exposed to ≥ 800 ppm CO_2_ and after 2 h a second set of thermal images was taken. Control plants included in the experiments were *ht1-2* (Hashimoto *et al.*, 2006), *ost1-3* and wild-type Col-0. Thermal images were converted into Flexible Image Transport System format (fits) using the ExaminIR software (FLIR). For the screen using the 96-pot-flat format, the temperature of plant leaves and the surrounding soil were measured using Fiji (Schindelin *et al.*, 2012). The soil temperature served as a location-specific reference to compensate for temperature variation depending on the position in the 96-pot flat. Either the temperature difference between plant leaves and surrounding soil or the average temperature of plant leaves was used as a quantitative measure. Plants with more than 1 °C difference from soil were considered as primary candidates and subject to further testing. The high-temperature leaf phenotype of *ht1-2* was used as a reference for CO_2_ insensitivity. To test the reproducibility of the CO_2_-dependent leaf temperature phenotype of putative candidates, T2 plants were grown in triplicate and assayed again alongside with *ht1-2* and wild-type control plants.

### Identification of amiRNA sequences and testing reproducibility

Genomic DNA from candidates with a robust and reproducible phenotype was isolated as described elsewhere (Edwards *et al.*, 1991) and the sequence of the amiRNA present was determined by sequencing of the PCR product (primers pha2804f and pha3479r; see Supplementary Table S1 at *JXB* online). Using the Target Search function available on the WMD3 website (Ossowski *et al.*, 2008), putative amiRNA target genes were predicted. For independent confirmation of the phenotype, independent lines were generated by cloning the identified amiRNA into pFH0032 (Supplementary Table S2; (Hauser *et al.*, 2013) and transforming it into Arabidopsis Col-0. Confirmed phenotypes were further analysed by using single knock-out mutants, higher order mutants generated by crossing and/or generating amiRNAs that target subsets of the initial target genes (see Supplementary Table S3).

### Gas exchange analyses

Stomatal conductance of H_2_O (*g*_s_) was measured in leaves of 5- to 6-week-old plants using portable gas exchange systems (LI-6400 and LI-6800, LI-COR, Lincoln, NE, USA), starting 2 h after growth chamber light onset. For intact single leaf ABA treatments, a LED light source set at 150 μmol m^−2^ s^−1^ (10% blue) and a chamber temperature of 21 °C was used. Leaves were equilibrated for 1 h at a relative humidity of 70–72%, airflow of 200 rpm and CO_2_ concentration of 400 ppm. After 1 h, steady-state stomatal conductance was recorded 10 min before the addition of ABA to the petioles submerged in water at the indicated concentration. For light-response measurements, plants were kept in the dark for 18 h prior to experiments. Stomatal conductance of a single intact leaf in the dark was recorded for 10 min, followed by red light treatment of 600 μmol m^−2^ s^−1^. After 20 min of red light treatment, additional blue light was applied at 10 μmol m^−2^ s^−1^. The incoming air humidity was kept at 62–65% and air flow at 200 rpm. For stomatal conductance measurements of single intact leaf CO_2_ responses, incoming relative air humidity was kept at 62–65% and the imposed changes in CO_2_ concentration were applied as indicated. Leaves were attached to intact plants and were equilibrated for 1 h before the measurements. The data presented represent *n*≥3 leaves with each leaf from independent plants per genotype per treatment.

### qRT-PCR analysis

Total RNA (500 ng) was reverse transcribed using the first-strand cDNA synthesis kit (GE Healthcare). qRT-PCR analyses were performed using 3-fold-diluted cDNA (Maxima SYBR Green Rox/qPCR Master Mix; Thermo Fisher Scientific) on a CFX Connect PCR cycler (BioRad). The housekeeping *PDF2* gene was used as an internal control (Czechowski et al 2005). The threshold cycle (*C*_T_) was determined by the instrument (CFX Manager Software, BioRad), and the ΔΔ*C*_T_ method was used to calculate the fold change in each gene (Livak and Schmittgen, 2001). For *RAB18* gene expression measurements, total RNA was extracted from 2-week-old seedlings that were treated with ABA for 9 h and final concentration of 20 µM.

## Results and discussion

### Generation of amiRNA library plants

We have previously described the generation of an amiRNA library consisting of 10 sublibraries that represent 22000 individual amiRNA designs (Hauser *et al.*, 2013). Deep sequencing of these 10 sublibraries showed that ≥95% of the designed amiRNAs were present in these sublibraries (Hauser *et al.*, 2013). The amiRNA library was transformed first into *Agrobacterium tumefaciens* and then into Arabidopsis Col-0. Over a period of over 4 years, the amiRNA library consisting of 10 sublibraries was transformed and T1 seeds harvested. Using plate- or soil-based selection methods, herbicide-resistant T1 plants were grown and T2 seeds from over 14000 individual plants were harvested (Table 1). The transformation rate varied over a range from 0.08% to 0.76% with an average of 0.25%. During the course of this research, approximately 3000 additional T2 lines (Zhang *et al.*, 2018) were generated expressing amiRNAs that target homologous transporter-encoding gene family members. These 3000 lines will also be made available to the ABRC, such that in the end over 14000 total T2 lines will be submitted for use by the community.

**Table 1.** Overview of the 10 amiRNA libraries as described by Hauser *et al*. (2013), the number of amiRNAs designed for each library, and the number of individual T2 amiRNA transformants that were generated here

Pool name	Pool description	AmiRNA designs	T2 lines
Kinase receptor (PKR)	Protein kinases, protein phosphatases, receptors and their ligands	1860	817
Binding (BNO)	Proteins binding small molecules	1968	1771
Protein (CSI)	Proteins that form or interact with protein complexes including stabilization of those	2313	842
RNA DNA (TFB)	Transcription factors and other RNA and DNA binding proteins	2964	831
Metabolism (TEC)	Metabolic and other enzymes catalysing transfer reactions (EC: 2)	1521	1548
Diverse enzymes (PEC)	Catalytically active proteins, mainly enzymes	1881	1113
Non-classified (UNC)	Genes for which the function is not known or cannot be inferred	4082	1387
Diverse molfun (DMF)	Protein with diverse functional annotation not found in the other categories	1505	1152
Hydrolase (HEC)	Hydrolytic enzymes (EC class 3), excluding protein phosphatases	2129	971
Transporter (TRP)	Proteins that transport molecules across membranes	1777	3844
Total			14276

Note that for the generation of each pool the 20 bp and 21 bp libraries were combined (see Hauser *et al*., 2013 for details). All T2 lines have been submitted to the Arabidopsis Biological Resource Center (ABRC).

### Screen for ABA-insensitive seed germination phenotype

**Fig. 1.**
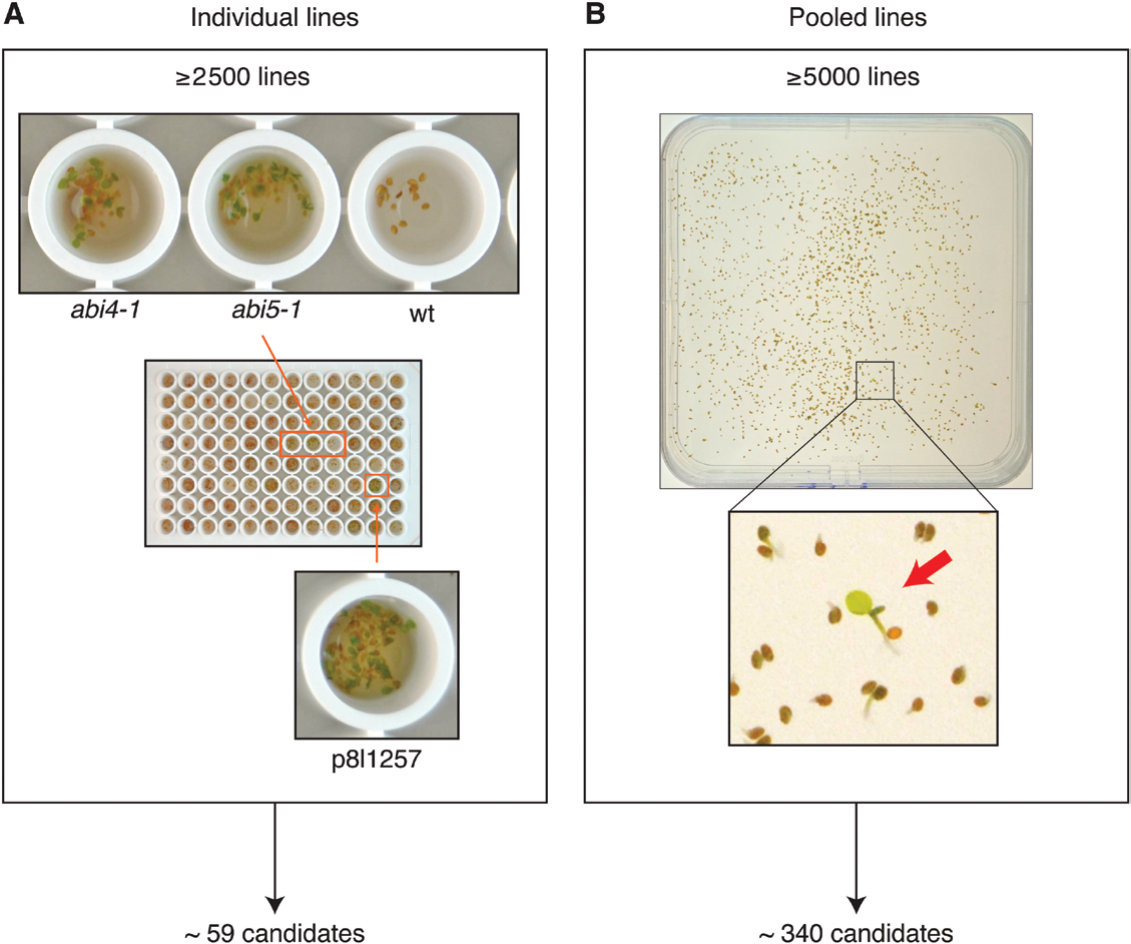
Overview of primary screen performed with over 7500 T2 amiRNA lines. (A) Approximately 2500 T2 amiRNA lines were screened individually by adding seeds to 96-well plates each well containing 100 µl 0.1% agarose supplemented with 2 µM ABA and approximately 10–20 seeds from one plant. Wild-type (Col-0), *abi4-1*, and *abi5-1* were used as controls. Based on visual inspection of cotyledon greening, around 59 lines were considered as candidates for further testing in the T3 generation. (B) Approximately 5000 T2 amiRNA lines were screened in pools. Each pool contained 10–50 seeds from 90 individually stored amiRNA lines (see main text). Approximately 340 lines were selected for further testing in the T3 generation.

In total over 2500 T2 amiRNA lines were screened individually and over 5000 T2 amiRNA lines were screened in pools for ABA-insensitive germination phenotypes (Fig. 1). In the primary screen using individual plants in a 96-well plate format, 59 putative candidates were identified. In the primary screen using pools of 90 plants with 25–80 seeds per line, approximately 340 putative candidates representing an unknown number of lines were identified (Fig. 1).

**Fig. 2.**
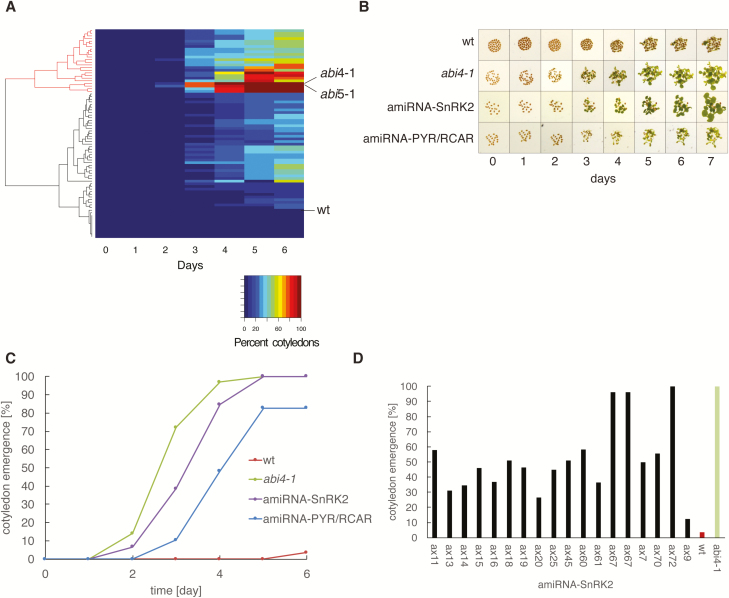
Overview of secondary ABA insensitivity screen performed with T3 candidate amiRNA lines identified in the primary screen that targets known genes involved in ABA signal transduction. (A) Heat map representation of cotyledon emergence time course of the T3 generation obtained from candidates with putative ABA-insensitive seed germination or cotyledon emergence phenotypes. Each row represents the percentage of cotyledon emergence of one individual line. The rows are ordered by hierarchical clustering. Wild-type control (WT, Col-0) and *abi4-1* and *abi5*-1 as reference for ABA insensitivity are shown. (B) Images of seeds germinating on plates in secondary screen containing 2 µM ABA at the indicated time points. Shown are representative plants of amiRNA plants targeting a set of six *SnRK2* kinase genes (*amiRNA-SnRK2*) or a set of three *PYR*/*RCAR* ABA receptor genes (*amiRNA-PYR*/*RCAR*; see Table 2). Wild-type control (Col-0) and *abi4-1* and *abi5-1* as reference for insensitivity are shown in the other two rows. (C) Time course of cotyledon emergence in the presence of ABA for wild-type control (wt), *abi4-1* as a reference for ABA insensitivity and two representative amiRNA lines that target a set of six *SnRK2* protein kinase genes (*amiRNA-SnRK2*) or a set of three *PYR*/*RCAR* ABA receptor genes (*amiRNA-PYR/RCAR*). Per genotype 74 ± 46 seeds were phenotyped. (D) Bar plot of variation of cotyledon emergence phenotype (day 6; 2 µM ABA) in the T3 generation of plants isolated in the primary screen that were selected as candidates based on their seed germination phenotype in the T2 generation. Sequencing of the amiRNA confirmed that all 18 of these amiRNA-expressing plants contain an amiRNA that targets a set of six *SnRK2* kinase genes (*amiRNA-SnRK2*; see Table 2). Wild-type control (wt, Col-0) and *abi4-1* as reference for ABA insensitivity are shown.

**Fig. 3.**
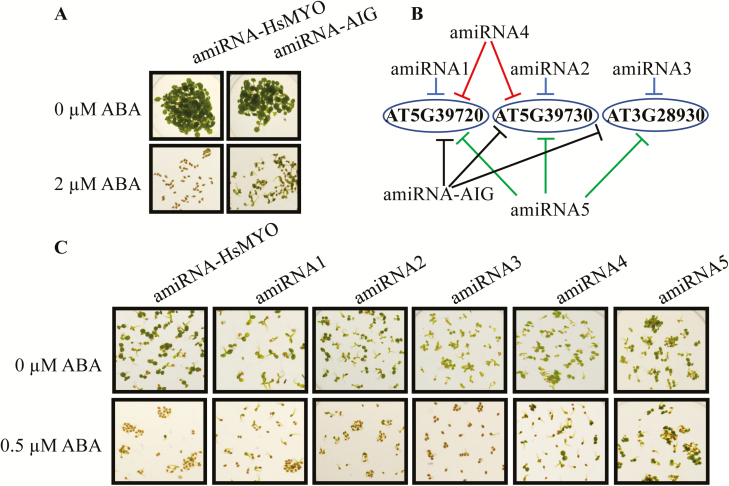
Avirulence-induced genes (*AIG2*s) targeted by an amiRNA cause a reduced ABA sensitivity in cotyledon emergence assays. (A) Seedlings of the control amiRNA line, which has no target gene in Arabidopsis (*amiRNA-HsMYO* control), and an amiRNA line that targets three *AIG2* genes (*amiRNA-AIG*) germinated in the presence of 0 or 2 µM ABA. Photographs were taken after 12 d of exposure. (B) Five new amiRNAs were designed to target the three *AIG2(-like*) genes. Three of these amiRNAs, amiRNA1, 2 and 3, target a single gene each. AmiRNA4 targets two tandem-repeat *AIG2(-like*) genes and amiRNA5 targets all three genes at non-identical nucleotides compared with the original amiRNA isolated in the primary screen (*amiRNA-AIG*, see Supplementary Table S2 for amiRNA sequences). (C) The new T2 amiRNA lines were tested in cotyledon emergence assays. Seedlings were germinated in the presence of 0 or 0.5 µM ABA. Photographs were taken after 4 d of incubation.

These candidates were subjected to further analysis in a secondary screen (Fig. 2). The cotyledon emergence phenotype of 24 T3 seedlings from a total of 76 retested plants showed a more reduced ABA sensitivity that was clearly different from wild-type and less severe than the *abi4-1* and *abi5-1* controls (Fig. 2A). From the 59 putative candidates identified using the individual screening approach, the amiRNA line p8l1257 showed a reproducible partial insensitivity to ABA in the T3 generation (Fig. 3). Only the amiRNA in candidates with the most robust phenotypes was determined by sequencing. Two of the amiRNA-targeted gene sets identified in 24 seedlings with reproducible phenotypes are known core components of the ABA signal transduction cascade (Fig. 2; Table 2). These include amiRNA lines that target the three ABA receptors PYR1 (RCAR11), PYL4 (RCAR10), and PYL6 (RCAR9) (Fig. 2B, C; Table 2). Furthermore, amiRNA-expressing plants that target six members of the SnRK2 protein kinase family (Mustilli *et al*., 2002; Yoshida *et al.*, 2002; Fujii and Zhu, 2009) were isolated in this screen, including the three SnRK2 protein kinases, SnRK2.2, SnRK2.3, and SnRK2.6 (OST1), that are known to be required for abscisic acid signaling (Fig. 2B, C; Table 2; Mustilli *et al*., 2002; Yoshida *et al.*, 2002; Fujii and Zhu, 2009).

**Table 2.** AmiRNA sequences and predicted target genes found in candidate T3 plants showing robust ABA-insensitive seed germination phenotypes in the T2 screen and subsequently in the T3 generation

No. of sequenced T3 plants	AmiRNA sequence	Target gene	Gene name
18	TGGATATGCTCCAACCGGCAT	AT1G10940	*SNRK2.4*
		AT1G60940	*SNRK2.10*
		AT2G23030	*SNRK2.9*
		AT3G50500	*SNRK2.2*
		AT4G33950	*SNRK2.6* (*OST1*)
		AT5G66880	*SNRK2.3*
5	TATCAACGACGTAAGACTCGT	AT2G38310	*PYL4* (*RCAR10*)
		AT2G40330	*PYL6* (*RCAR9*)
		AT4G17870	*PYR1* (*RCAR11*)
1	TTAATACATGGATGCACACGT	AT3G28930	*AIG2*
		AT5G39720	*AIG2LA*
		AT5G39730	*AIG2LB*

Notably, Fig. 2D shows a strong variation in the cotyledon emergence phenotype among plants expressing the same amiRNA that targets six *SnRK2* kinase transcripts. This variation might be responsible for the high number of variable candidates that did not show a robust phenotype following the primary screen. Additional amiRNA lines were isolated as putative mutants and the amiRNA sequence was determined (Supplementary Table S4). Although some of the predicted targets might be expected to affect abscisic acid responses, rescreening of these putative mutants did not show consistently robust reproducible phenotypes. Thus, amiRNAs appear to produce phenotypes that may be variable even within the same line. These findings led us to develop a protocol in which: (i) only putative mutants that showed a consistent phenotype when screening seeds from the next generation of plants were selected, and (ii) Only lines that showed similar phenotypes upon re-transformation with new amiRNAs that are predicted to target the same genes were selected. Furthermore, based on the variation observed here in the secondary screen it is advisable to investigate over 10 independent transformed lines (Schwab *et al.*, 2006; Hauser *et al.*, 2013) in the future to determine which amiRNAs produce phenotypes that can be carried forward. The isolation of amiRNA lines targeting functionally overlapping *PYR*/*RCAR* ABA receptor and *SnRK2* protein kinase genes, which could not be isolated in traditional forward genetic mutant screens, provides a proof of principle that functionally redundant genes can be isolated in forward genetic screening using this new amiRNA resource. The inclusion of control lines and the validation steps described above should enable screening for diverse phenotypes using the lines generated here that are being provided to ABRC.

### AmiRNA lines targeting three avirulence-induced genes show partial insensitivity to ABA inhibition of seed germination but not to ABA-induced stomatal closure

The amiRNA in line p8l1257 isolated in the present screen targets a new set of three genes (Fig. 3A, 3B). Previous research annotated these genes based on their mRNA upregulation in a transcriptomic study after infection with *Pseudomonas syringae* pv *maculicola* carrying avrRpt2 (avrRpt2-induced gene, *AIG2*) (Reuber and Ausubel, 1996). However, these genes have not been previously described to be involved in ABA-mediated control of seed germination or other phenotypic responses in plants.

The line p8l1257 was named *amiRNA-AIG* here and was further tested by analysing seed germination with additional T2 generation seeds from the original p8l1257 stock (Fig. 3). Germination properties were compared with a control amiRNA line targeting the human myosin 2 (*amiRNA-HsMYO*), which has no targeted genes in Arabidopsis (Hauser *et al.*, 2013). After 12 d on plates containing 2 µM ABA, the *amiRNA-AIG* line showed cotyledon greening in contrast to the control *amiRNA-HsMYO* line (Fig. 3A). The effect of the *amiRNA-AIG* on the expression of a known ABA-induced gene, *RAB18*, was analysed by qRT-PCR (see Supplementary Fig. S1). The ABA-mediated induction of *RAB18* expression was substantially reduced in the *amiRNA-AIG* line indicating a role of the targeted avirulence-induced genes (*AIG2s*) in ABA signal transduction.

Since two out of the three genes are tandemly repeated, generation of double mutants using T-DNA insertion knockouts would be limited. Therefore, five new amiRNA lines were generated that target subsets of genes targeted by the original *amiRNA-AIG* to verify the relevance of the predicted *AIG2* target genes. *AmiRNA1*, *2* and *3* targeted each a single *AIG2* (Fig. 3B; Supplementary Table S3). *AmiRNA4* targeted two tandem-repeat *AIG2* genes and *amiRNA5* targeted all three *AIG* genes targeted in the original *amiRNA-AIG* line, but with a different amiRNA sequence (Fig. 3B; Supplementary Table S3 for amiRNA sequences). When the T2 seeds expressing these five new amiRNAs were tested in a seed germination assay with 0.5 µM ABA, only the *amiRNA4* and *amiRNA5*-expressing lines showed less sensitivity to ABA compared with the control *amiRNA-HsMYO* line in cotyledon greening (Fig. 3C). The expression of all three putative target genes (AT5G39720, AT5G39730, AT3G28930) was analysed using qRT-PCR in the originally isolated *amiRNA-AIG* line and in all the *amiRNA* lines 1–5 (see Supplementary Fig. S2). The amiRNA efficiency of transcriptional inhibition varies between the lines, target transcript(s) and amiRNA sequence. Note that microRNA silencing in plants occurs via two mechanisms, (i) the degradation of transcripts and (ii) inhibition of translation (Brodersen *et al.*, 2008). Thus, quantification of targeted transcripts may not fully show the degree of silencing of target genes. Combined, these data provide evidence that the original *amiRNA-AIG* phenotype is attributable to silencing of more than one *AIG2* gene, suggesting overlapping homologous gene functions.

**Fig. 4.**
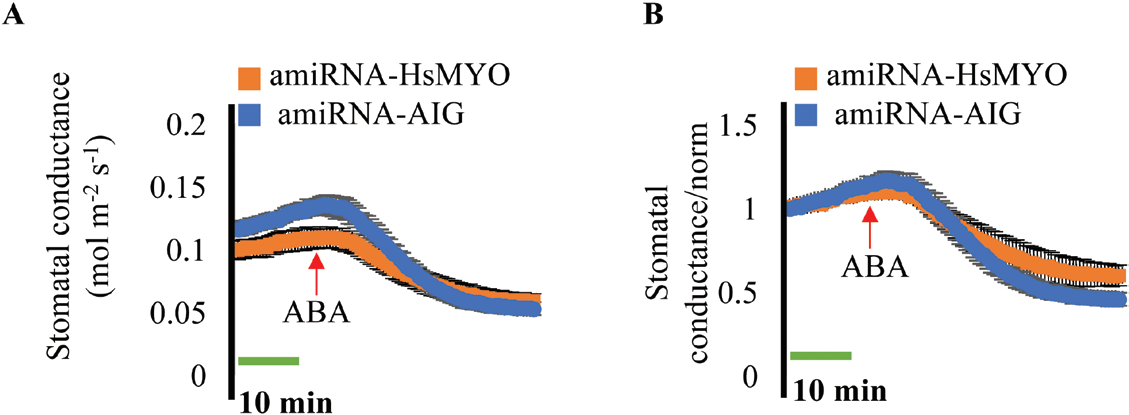
The isolated amiRNA line targeting three *AIG2* genes (*amiRNA-AIG*) responds to ABA in whole leaf gas exchange analyses. Time-resolved stomatal conductance to H_2_O (*g*_s_) in response to application of 2 µM ABA to the transpiration stream (red arrows) is shown in the amiRNA control line (*amiRNA-HsMYO*) and in the amiRNA line targeting three *AIG2* genes (*amiRNA-AIG*). Stomatal conductance was analysed using whole leaf gas exchange. (A) Stomatal conductance in mol m^−2^ s^−1^. (B) Data from (A) were normalized to stomatal conductance at the beginning of the experiment. Data are the mean of *n*=3 leaves per genotype ±SEM.

The original *amiRNA-AIG* line was also investigated to determine if it affects ABA-induced stomatal closure using an intact leaf gas exchange analysis approach. When ABA was applied to the transpiration stream of intact leaves at a final concentration of 2 µM, both the control *amiRNA-HsMYO* line and the *amiRNA-AIG* line showed an ABA-induced decrease in stomatal conductance to H_2_O (*g*_s_, Fig. 4A). Normalization of the stomatal conductance data showed no dramatic difference in ABA-induced stomatal closure between *amiRNA-HsMYO* and *amiRNA-AIG* (Fig. 4B). Together, the present data show that the isolated *amiRNA-AIG* line is less sensitive to ABA inhibition of seed germination.

The *AIG2* genes are functionally annotated as putative γ-glutamyl cyclotransferases (GGCTs, EC:4.3.2.9) based on their similarity to the human orthologue (HsGGCT; O75223). AIG2LA and AIG2LB share only 16% and 17% identity, respectively, to the human orthologue. GGCTs have been described to cleave γ-glutamyl-amino acid dipeptides to release the free amino acid and 5-oxoproline (Oakley *et al.*, 2008). Further research will be required to determine the mechanism by which AIG2s affect ABA inhibition of seed germination.

### Screen for CO_2_-insensitive leaf temperature phenotype

**Fig. 5.**
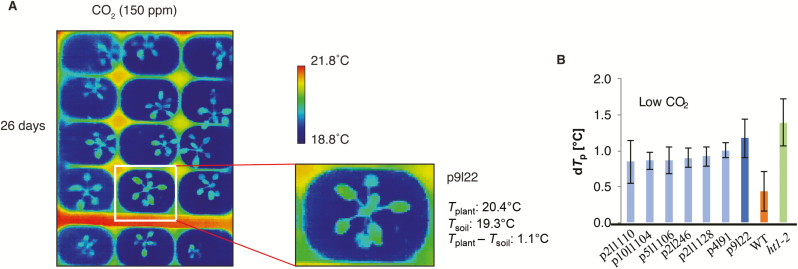
Thermal imaging screen for mutants with impaired response to low CO_2_ identified the amiRNA line *p9l22*. (A) Thermal images of amiRNA lines in the primary screen after exposure to low CO_2_ (150 ppm). Plants were grown in 96-pot flats under ambient CO_2_ and after 26 d exposed to low (150 ppm) CO_2_ for 2 h and then thermal images of the entire flat were taken in eight separate images per flat. An image is shown from the primary screen in which the plant *p9l22* (white box) was flagged as a candidate with a putative altered response to CO_2_ based on the leaf temperature. The average leaf temperature was computationally calculated across all rosette leaves for flagging putative mutants. (B) Differences of leaf temperature (*T*_plant_) and surrounding soil (*T*_soil_) for putative mutants exposed to low [CO_2_] (150 ppm) for 2 h. Average leaf temperature was computed by image analysis of the most fully expanded rosette leaves. Bars show average ±SD (*n*=3–5 leaves). WT (Col-0) (orange) and *ht1-2* (green) were used as control.

In total, over 2500 T2 amiRNA lines were screened individually for an altered leaf temperature response to a low CO_2_ concentration (150 ppm) by infra-red thermal imaging (Fig. 5). Leaf temperature depends on various parameters including radiation absorption, air temperature, and humidity (Merlot *et al.*, 2002). Low ambient CO_2_ concentration leads to stomatal opening in Arabidopsis, causing an increased transpiration rate and thus a decrease in leaf temperature compared with the surrounding air. Mutants impaired in CO_2_-induced stomatal opening appear warmer compared with wild-type plants. In the screen, we used the soil temperature as reference to compensate for the local temperature differences due to various factors including humidity of the soil. Wild-type plants and plants of the HIGH LEAF TEMPERATURE1-2 (*ht1-2*) mutant (Hashimoto *et al.*, 2006) were included in all trays as controls. Based on visual inspection of the thermal images, plants with relatively higher leaf temperature under low [CO_2_] compared with the other plants in the same image were selected and the difference between the average leaf temperature and the surrounding soil was determined. The difference between leaf temperature and soil temperature was determined as reference for overall temperature and to compensate for local temperature differences. A set of 106 plants with more than one degree difference between the leaf temperature and the surrounding soil was defined as initial putative candidates for further testing (see Methods for details). For the rescreening of putative mutants, we set a high threshold for temperature differences in the selection of mutants compared with the wild-type strain of 1 °C. The constitutive CO_2_ response mutant *ht1-2*, when exposed to low [CO_2_], shows a delta temperature above 1 °C between leaf and soil. Rescreening of these candidates in the T2 generation revealed an amiRNA line (*p9l22*) with a robust and reproducible impaired response to low CO_2_ (Fig. 5B).

**Fig. 6.**
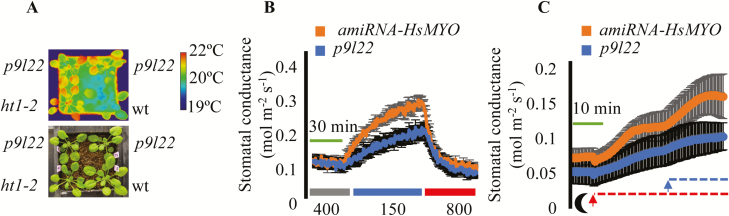
The amiRNA line *p9l22* is defective in light- and low-CO_2_-induced stomatal opening. (A) The *p9l22* line shows an elevated leaf temperature phenotype at low ambient [CO_2_] treatment (150 ppm). Top, thermal imaging; bottom, image of the same plants. The calibration bar shows the pseudo-colored scale for temperature. (B) Time-resolved stomatal conductance responses at the imposed [CO_2_] shifts (bottom in ppm) in the control *amiRNA-HsMYO* line and in the *p9l22* line were analysed using intact whole-leaf gas exchange. The plots represent average ±SEM (*n*=4 leaves from four plants per genotype). (C) Time-resolved stomatal conductance responses from darkness to the imposed light intensity and light quality shifts (bottom moon shape: darkness; red dashed line: red light at 600 μmol m^−2^ s^−1^; and blue dashed line: blue light at 10 μmol m^−2^ s^−1^) in the control *amiRNA-HsMYO* line and in the *p9l22* line were analysed using intact whole-leaf gas exchange. The plots represent average ±SEM (*n*=3 leaves from three plants per genotype).

After exposure to low [CO_2_], the leaf temperature of the *p9l22* line was compared with wild-type (Col-0) and with the constitutive high-CO_2_-response mutant *ht1-2* (Fig. 6A; Hashimoto *et al.*, 2006). The leaves of the *p9l22* line had a higher temperature than wild-type leaves and a similar temperature to *ht1-2* leaves (Fig. 6A). Stomatal index (SI) and density (SD) were calculated for wild-type, the control *amiRNA-HsMYO*, and *p9l22* lines. No noteworthy differences were found between the genotypes (Supplementary Fig. S3; *amiRNA-HsMYO* versus *p9l22* line, one-way ANOVA, *P*>0.05 for SI and SD).

To measure [CO_2_] responses in a time-resolved fashion, we measured stomatal conductance (*g*_s_) using a gas exchange analyser. In the *amiRNA-HsMYO* control line, the shift from ambient (400 ppm) to low (150 ppm) [CO_2_] led to a rapid increase in stomatal conductance (Fig. 6B). *AmiRNA* line *p9l22* responded to the same treatment with a lower magnitude of stomatal opening (Fig. 6B). Both lines showed stomatal closure in response to high (800 ppm) [CO_2_] exposure at similar rates (Fig. 6B). To test whether line *p9l22* is defective in response to other stimuli that cause stomatal opening, light-induced *g*_s_ responses were investigated (Fig. 6C). The control *amiRNA-HsMYO* and *p9l22* lines were kept in the dark for 18 h prior to the experiments and steady-state *g*_s_ was measured. When red light (at 600 µmol m^−2^ s^−1^) was applied, the *p9l22* line showed a reduced rate of *g*_s_ increase when compared with the control line. The same was observed when blue light (at 10 µmol m^−2^ s^−1^) was superimposed on the red light background (Fig. 6C). Thus, the amiRNA line *p9l22* causes reduced responses to low CO_2_ concentration, red light, and blue light.

The amiRNA in the *p9l22* line was sequenced and is predicted to target two homologous proteasomal subunit genes (*PAB1*, AT1G16470; and *PAB2*, AT1G79210). *PAB1* and *PAB2* are the sole genes that encode the 20S proteasome α2 subunit (Baumeister *et al.*, 1998). First, we attempted to isolate a double mutant (*pab1 pab2*) using T-DNA insertion lines (SALK_099950 and SALK_144987; Alonso *et al.*, 2003). After genotyping over 100 plants in the F2 generation, no homozygous double mutant was recovered. We concluded that the double mutant is very likely lethal.

**Fig. 7.**
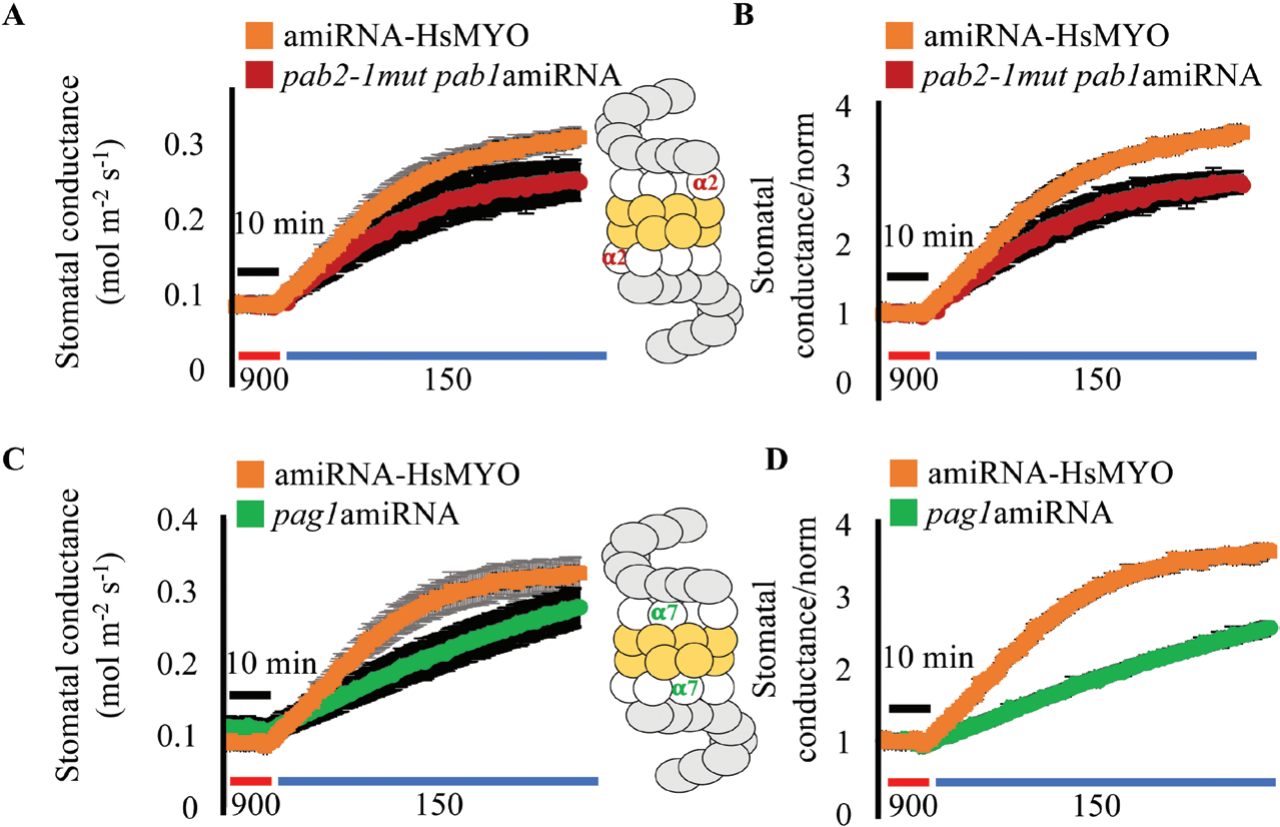
New amiRNA lines targeting the two *PAB* genes (α2 subunit) and *PAG1* gene (α7 subunit) of the 26S proteasome show partial impairment in low-CO_2_-induced stomatal opening. (A, C) Time-resolved stomatal conductance responses at the imposed [CO_2_] shifts (bottom in ppm) in the control line *amiRNA-HsMYO* and (A) in the *pab2-1mut pab1amiRNA* line (*pab2* gene T-DNA knockout and *pab1* gene amiRNA knockdown, α2 subunit) and (C) *amiRNA-PAG1* line (*pag1* gene amiRNA knockdown, α7 subunit) were analysed using intact whole-leaf gas exchange. The plots represent average ±SEM (*n*=3–4 leaves from different plants per genotype). (B, D) Data from (A, C) were normalized to the average stomatal conductance of the first 10 min. Inserts show a representation of the 26S proteasome, with the α2 subunits highlighted in red and α7 subunits highlighted in green. The initial slope of *g*_s_ response for *amiRNA-HsMYO* and *amiRNA-PAG1* was calculated and one-way ANOVA was used to compare the values (*P*>0.05).

Alternatively, a new amiRNA sequence targeting solely the *PAB1* gene was cloned and transformed into the *pab2-1* single mutant (SALK_144987). This new amiRNA line, *pab2-1*mut *pab1amiRNA*, was investigated in stomatal conductance analyses of [CO_2_] responses (Fig. 7). Leaves were first exposed to high (900 ppm) [CO_2_] for 1 h and steady-state *g*_s_ was recorded. Shifts to low (150 ppm) [CO_2_] led to an increase in *g*_s_ in both the *pab2-1*mut *pab1amiRNA* line and the control *amiRNA-HsMYO* line (Fig. 7A). The normalized stomatal conductance data show that the *pab2-1 amiRNA-PAB1* line responds to low [CO_2_] with a reduced magnitude compared with the control line (Fig. 7B).

Initial experiments were pursued to determine if modifications in the α-ring of the 20S proteasome might be linked to the above phenotypes, or whether this mutation is specific to only α2 subunit mutations of the proteasome. The α-ring of the 20S proteasome is composed of seven α-subunits (Kurepa and Smalle, 2008). The *p9l22 amiRNA* targets the only two genes that encode the α2 subunit of the proteasome (Fig. 7, inset highlighted in red). To determine whether other α-subunits also affect the response to low [CO_2_], a second amiRNA line was generated, which targets the *PAG1* gene (α7 subunit, inset in Fig. 7 highlighted in green), named *amiRNA-PAG1*. The α7 subunit is encoded by a single gene in Arabidopsis (Kurepa and Smalle, 2008). When a *amiRNA-PAG1* line was tested in *g*_s_ responses to [CO_2_] shifts, it showed a lower rate of stomatal opening when compared with the control *amiRNA-HsMYO* line (Fig. 7C, D; one-way ANOVA *P*>0.05). The expression levels of *PAB1*, *PAB2*, and *PAG1* were analysed in the *p9l22* amiRNA line and also in *pab2-1*mut *pab1amiRNA* and *amiRNA-PAG1* lines using qRT-PCR (see Supplementary Fig. S4). With the exception of the severely reduced *PAB2* expression in the *pab2-1*mut *pab1amiRNA* line when compared with control lines, no clear evidence for knock down at the transcriptional level could be detected in the amiRNA lines, which may point to amiRNA-mediated inhibition of translation (Brodersen *et al.*, 2008).

The present findings show that the *p9l22* amiRNA line is also partially impaired in red light-induced stomatal opening. Red light mediates stomatal opening in part via activation of photosynthesis and the resulting drop in internal concentration of CO_2_ (*C*_i_) (Roelfsema *et al.*, 2002; Matrosova *et al.*, 2015). In addition, the *p9l22* line is also partially impaired in blue light-induced stomatal opening. This suggests that a general regulator of stomatal opening is impaired in this line. As the proteasome mediates the degradation of proteins and reduced functions of α-ring subunits are predicted to increase protein levels, it is tempting to speculate that the phenotype observed might be correlated with an increased abundance of a negative regulator of stomatal opening. Further research will be required to test this or other hypotheses. In other studies, the 26S α2 subunit, when overexpressed, enhanced thermotolerance and adaptation in rice and Arabidopsis, suggesting that proteasomal subunits can have rate-limiting roles in regulating plant physiological responses (Li *et al.*, 2015).

### Summary and future use

A library of over 11000 plus 3000 additional T2 generation amiRNA lines was created as a new resource to screen the redundant gene space in Arabidopsis. These amiRNA-expressing lines are being provided as individual lines to the Arabidopsis Biological Resource Center (ABRC). Given the observations and findings in the present study, lines will be available for high-throughput screening in pools of 90 lines per pool with approximately 25–50 seeds per individual amiRNA line in each pool. In each pool, the pooled seeds for screening will originate from one of the 10 sublibraries that target gene family members with defined functional annotations (Table 1; Hauser *et al.*, 2013). This approach will increase the probability of identifying interesting putative mutants in future screens despite the biological variability in amiRNA silencing lines found here (Fig. 2D).

The screen for ABA-insensitive seed germination phenotypes identified two amiRNAs targeting *PYR*/*RCAR* ABA receptor genes and *SnRK2* genes, which are both known groups of redundant key genes and proteins required for ABA signal transduction (Ma *et al.*, 2009; Park *et al.*, 2009). Isolating amiRNA lines in these known components serves as proof of principle for our approach. Moreover, screening this amiRNA population enables the identification of mutants that require co-silencing of homologous gene family members, which are less likely to be found in forward genetic screens of ethyl methanesulfonate or T-DNA mutagenized seed populations. Overall the presented amiRNA screen shows that amiRNA lines are prone to showing a high rate of variable candidates with weak or non-robust phenotypes. Nevertheless, as shown here new mutants can be isolated. Furthermore, during the course of this research, this amiRNA library resource has been used to isolate long-sought functionally redundant auxin transporter genes (e.g. *ABCB6*, *ABCB20*; Zhang *et al.*, 2018). Approaches to circumvent the inherent limitations of forward genetic screening with amiRNAs were developed in the present study. As a first step, it is recommended to rescreen the next generation to identify robust and reproducible phenotypes in individually isolated putative mutant lines. As a second step, the amiRNA sequence of confirmed mutant lines needs to be determined (see Methods). AmiRNA sequences linked to the observed phenotypes are retransformed, and testing over 10 independent lines for the phenotype is recommended. Alternatively, amiRNA on one line without break that target a subset of the initially predicted targets can be used to narrow down the causative genes (e.g. Fig. 3). For cases where only two to three genes are targeted, T-DNA lines or CRISPR/Cas9 mutants can be used to narrow down the genes relevant for the phenotype.

Over 95% of the amiRNAs in this library were designed to target only two to five genes (Hauser *et al.*, 2013), meaning that identification of causal genes is facilitated. Using the above approach, we report on two newly identified mutants: (i) amiRNA lines targeting three genes encoding avirulence induced gene (2-like) proteins show an ABA-insensitive seed germination phenotype; and (ii) amiRNA lines targeting two proteasomal subunits show insensitivity to low-CO_2_-induced stomatal opening. Further analyses of the two targeted genes in this amiRNA line suggest that stronger T-DNA alleles result in lethality. This indicates the usefulness of the generated amiRNA lines for forward genetic isolation of higher order mutants that would be lethal upon knock-out. Our data suggest that the wild-type expression levels of two α-subunits of the 20S proteasome, α2 and α7, are required for fully functional stomatal opening mediated by physiological stimulation. This indicates that the proteasomal subunits are likely controlling an unknown general negative regulator of stomatal opening. The amiRNA seed resource generated here provides a new and potent tool to identify redundant genes and also lethality causing higher order mutants in many biological processes in Arabidopsis.

In conclusion, the amiRNA library resource is well suited for screening of phenotypes that can be easily verified in subsequent generations. This population may be best suited for screens that permit high throughput or medium throughput screening for phenotypes with a large dynamic range. Many such powerful screens have been performed in classical Arabidopsis mutant screens that were previously not designed to identify functionally redundant genes.

## Supplementary data

Supplementary data are available at *JXB* online.

Fig. S1. The induction of *RAB18* gene expression by ABA is lower in the *amiRNA-AIG* lines.

Fig. S2. The expression of *AIG2* genes in *amiRNA-AIG*s lines.

Fig. S3. The *p9l22* amiRNA line has normal stomatal indices and density.

Fig. S4. The expression of *PAB1*, *PAB2*, and *PAG1* genes in amiRNA lines.

Table 1. List of relevant primers used in this study.

Table 2. List of relevant plasmids used in this study.

Table 3. List of new amiRNAs designed and cloned in this study.

Table 4. AmiRNA sequences and predicted target genes found in candidate plants.

## Supplementary Material

Supplementary Material
