# Supplementary material for: A seed resource for screening functionally redundant genes and isolation of new mutants impaired in CO2 and ABA responses
Source: J Exp Bot. 2018 Oct 20;70(2):641–51. doi: 10.1093/jxb/ery363 (PMC6322574; doi:10.1093/jxb/ery363)
Supplement: Supplementary Material [file ery363_suppl_supplementary_data.pdf]

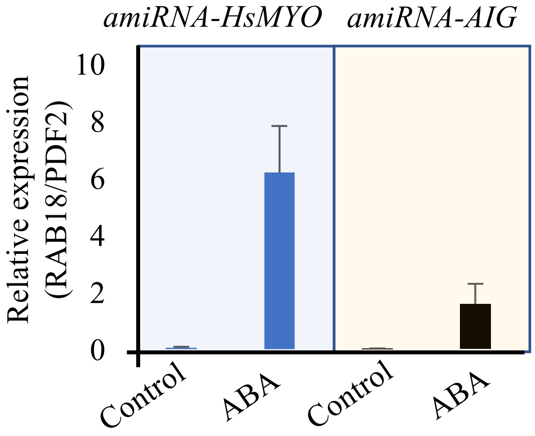

Figure S1: ABA-induction of *RAB18* gene expression is lower in the amiRNA-AIGs line. The expression of *RAB18* gene was measured in Arabidopsis seedlings (two-week-old) nine hours after ABA treatment (ABA) or ethanol (Control). qRT-PCR was performed using total RNA from seedlings and the *PDF2* gene expression was used as an internal control. The graph shows data from three biological replicates with two technical replicates each. Bars show mean ± SD.

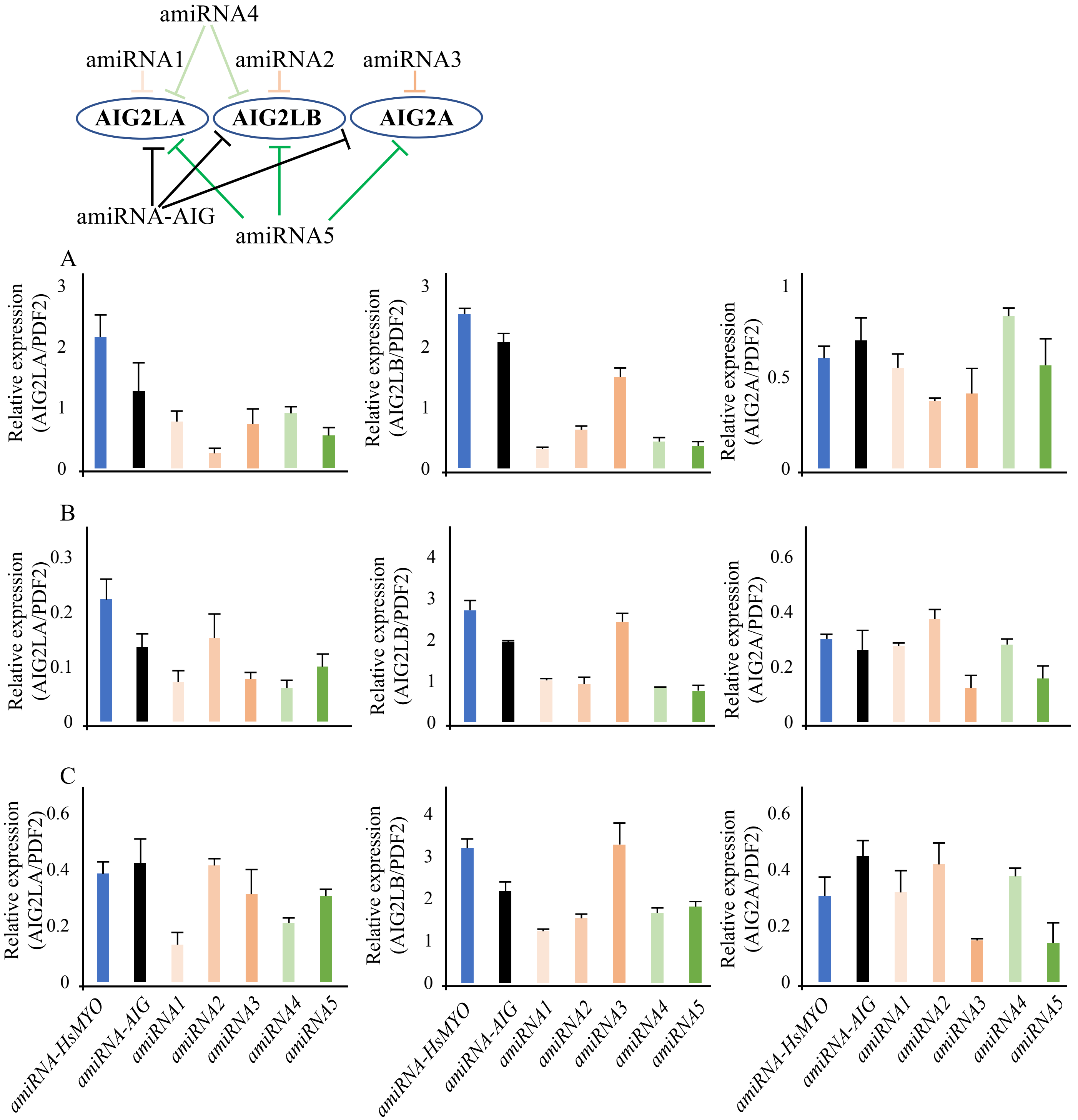

Figure S2: The expression of *AIG2* genes in amiRNA-AIGs lines. The expression of three *AIG2* genes was evaluated in *Arabidopsis* seedlings (two-week-old). qRT-PCR was performed using total RNA from seedlings. The *PDF2* gene expression was used as an internal control. Panels "A", "B" and "C" represent three biological replicates with three technical replicates for each biological replicate. AIG2LA (AT5G39720), AIG2LB (AT5G39730), AIG2A (AT3G28930).

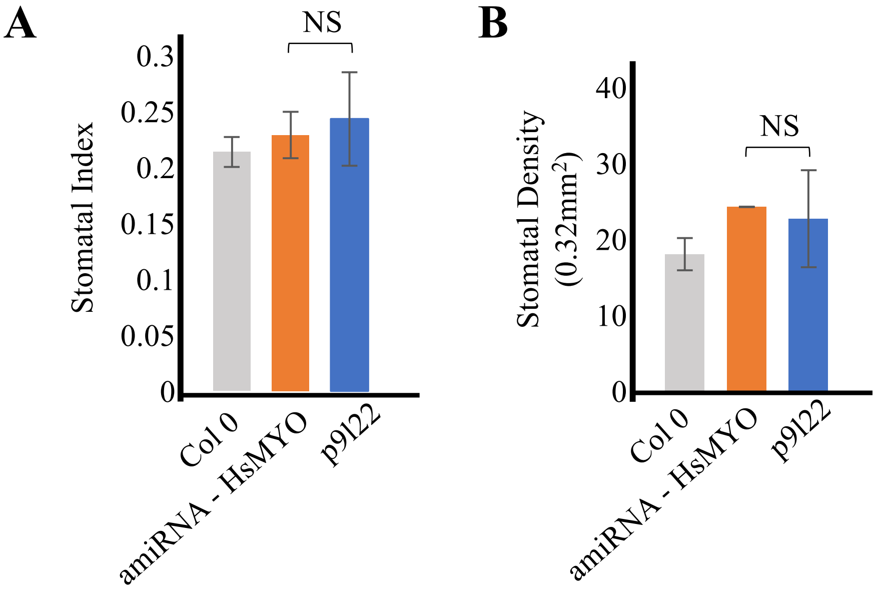

Figure S3. The *p9l22* amiRNA line has normal stomatal indices and density when compared to the *amiRNA-HsMYO* control line. Stomatal index **(A)** and stomatal density **(B)** measurements of the abaxial side of the fifth true leaf of 3 to 4-week-old plants. Wild type (Col 0), *amiRNA-HsMYO* and *p9l22* lines were evaluated. Data represent average of stomatal indices and density ± SD (n= 3 plants / three images per plant). NS, not significant (One-Way ANOVA $p>0.05$).

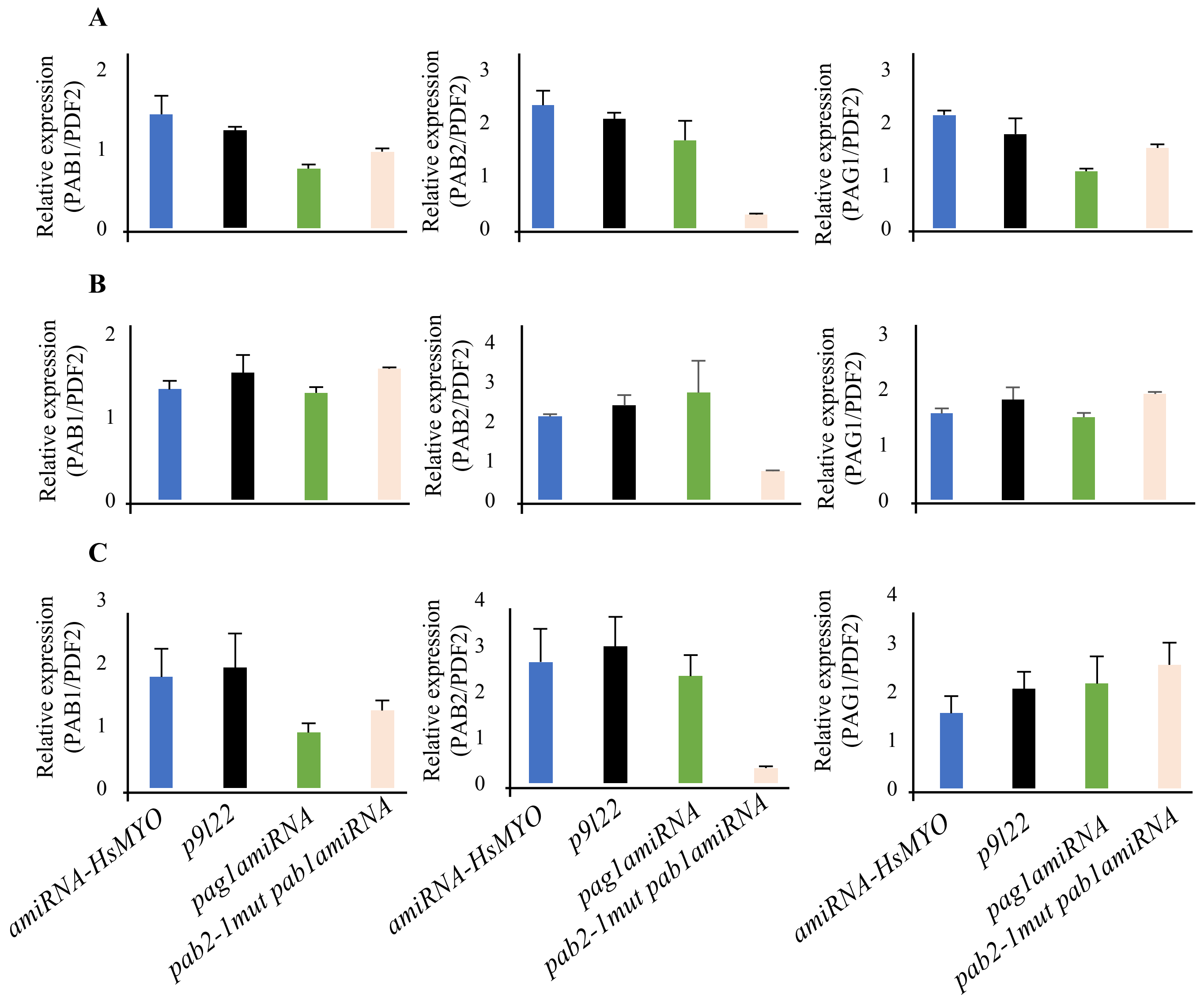

Figure S4: The expression of *PAB1, PAB2* and *PAG1* genes in amiRNA lines. The expression of three proteasomal subunits genes was evaluated in Arabidopsis seedlings (two-week-old). qRT-PCR was performed using total RNA from seedlings. The *PDF2* gene expression was used as an internal control. Panels "A", "B" and "C" represent three biological replicates with three technical replicates for each biological replicate. PAB1 (AT1G16470), PAB2 (AT1G79210), PAG1 (AT2G27020).

Supplemental Table 1. Comprehensive list of relevant primers used in this study.

Primer name	Sequence	*Reference or Source*
pha2804f	AGAGAACACGGGGGACGAG	This work
pha3479r	AAACCGGCGGTAAGGATCTG	This work
PIE1-ImiR-s	gaTAGGATTCCAGTCAGTCTCTAtctctcttttgtattcc	This work
PIE1-IImiR-a	gaTAGAGACTGACTGGAATCCTAtcaaagagaatcaatga	This work
PIE1-IIImiR*s	gaTAAAGACTGACTGCAATCCTTtcacaggtcgtgatatg	This work
PIE1-IVmiR*a	gaAAGGATTGCAGTCAGTCTTTAtctacatatatattcct	This work
dreb1_ImiR-s	gaTGCCACGTCGTGAGCACGCATtctctcttttgtattcc	This work
dreb1_IIImiR-a	gaATGCGTGCTCACGACGTGGCAtcaaagagaatcaatga	This work
dreb1_IIIImiR*s	gaATACGTGCTCACGTCGTGGCTtcacaggtcgtgatatg	This work
dreb1_IIVmiR*a	gaAGCCACGACGTGAGCACGTATtctacatatatattcct	This work
prxcb1-I_miR-s	gaTTTCCCAAAGGAACCGTCCAGtctctcttttgtattcc	This work
prxcb1-I_II_miR-a	gaCTGGACGGTTCCTTTGGGAAAtcaaagagaatcaatga	This work
prxcb1-I_III_miR-s	gaCTAGACGGTTCCTATGGGAATtcacaggtcgtgatatg	This work
prxcb1-I_IV_miR-a	gaATTCCCATAGGAACCGTCTAGtctacatatatattcct	This work

Supplemental Table 2. Comprehensive list of relevant plasmids used in this study.

Plasmids	***Description***	***Reference or Source***
pFH0032	$Spec^r$, Cam^r, pGreen derivative containing 35Sp gateway ocst	(Hauser et al., 2013)
pFH0332	$Spec^r$, pGreen derivative containing 35Sp amiRNA AT1G12610; AT2G35700; AT4G16750; AT4G32800; AT5G11590; AT5G25810 ocst	This work
pFH0333	$Spec^r$, pGreen derivative containing 35Sp amiRNA AT2G18140; AT2G18150; AT3G32980; AT3G49120 ocst	This work
pFH0334	$Spec^r$, pGreen derivative containing 35Sp amiRNA AT2G28290; AT3G12810 ocst	This work

Supplemental Table 3. Comprehensive list of new amiRNAs designed and cloned in this study.

Lines	***amiRNA sequence***
P8l1257/amiRNA-AIG	TTAATACATGGATGCACACGT
amiRNA1	TTATAGAACATACTCAGACAC
amiRNA2	TACAATAACCTAGCTAGACAC
amiRNA3	TTCTATACAAGTGATAGGCGT
amiRNA4	TTAATACATGGATACAGACGC
amiRNA5	TTAATACATGGATACAGACGA
P9l22	TGTAATCATCGATATTTGCTG
pab2mutpab1amiRNA	TCATTAGTTATTGAGAGGCAC
pag1amiRNA	TTTTTACTCGACTTCAGGCAA

Supplemental Table 4 AmiRNA sequences and predicted target genes found in candidate T3 plants which showed a putative ABA insensitive seed germination phenotype in the T2 screen and in the T3 generation.

Number of T3 plants	amiRNA sequence	Target genes	Gene name	Reproduced in independent transformants(1)
1	TAGGATTCCAGTCAGTCTCTA	AT3G12810	PHOTOPERIOD-INDEPENDENT EARLY FLOWERING 1 (PIE1); (chr13); (SRCAP)	No
		AT2G28290	CHROMATIN REMODELING COMPLEX SUBUNIT R 3 (CHR3); SPLAYED (SYD)	No
1	ATCTTGAGCGATTTGACGTTC	AT4G40030	Histone superfamily protein	NA
		AT1G75600	Histone superfamily protein	NA
		AT5G65360	Histone superfamily protein	NA
		AT5G10400	Histone superfamily protein	NA
		AT1G13370	Histone superfamily protein	NA
		AT5G65350	HISTONE 3 11 (HTR11)	NA
1	TTGAGGACATCTCCCAACCAA	AT2G44020	Mitochondrial transcription termination factor	NA
		AT2G21710	embryo defective 2219	NA
1	TTTCCCAAAGGAACCGTCCAG	AT2G18140	Peroxidase	No
		AT2G18150	Peroxidase	No
		AT3G32980	Peroxidase	No
		AT3G49120	PEROXIDASE CB (PRXCB)	No
1	TTAAAGACCATACTTACGCTT	AT3G02590	Fatty acid hydroxylase	NA
		AT3G02580	STEROL 1 (BR synthesis)	NA
5	TGCCACGTCGTGAGCACGCAT	AT4G32800	ERF/AP2 transcription factor	no
		AT4G16750	ERF/AP2 transcription factor	
		AT1G12610	DWARF AND DELAYED FLOWERING 1 (DDF1)	
		AT2G35700	ERF FAMILY PROTEIN 38 (ERF38)	
		AT5G25810	TINY	
		AT5G11590	TINY2	
1	TTTAGTCCTACCTTGAAACCT	AT2G39550 AT5G40280	Prenyltransferase family protein/ERA1	NA
1	TCATTACGATTTGTTTCGCTC	AT5G10100 AT1G35910	TREHALOSE-6-PHOSPHATE PHOSPHATASE	NA
1	TTACACTATGTCCTTGTTCTC	AT3G43670 AT2G06830 AT1G09970	Copper amine oxidase family protein	NA

[1] No: The amiRNA was cloned, transformed into Arabidopsis, independent lines were selected and the T2 seeds were tested for insensitivity to abscisic acid in seed germination. The cotyledon emergence phenotype was not clearly different from wild type i.e. the phenotype observed in the T2 screen and the assay with the T3 seeds could not be reproduced. NA: no independent lines were generated.

Supplemental references

Hauser, F., Chen, W., Deinlein, U., Chang, K., Ossowski, S., Fitz, J., Hannon, G. J., and Schroeder, J. I. (2013). A genomic-scale artificial microRNA library as a tool to investigate the functionally redundant gene space in *Arabidopsis*. *Plant Cell* **25**:2848–2863.
